# Nutritional Composition Assessment of 3000 Individualized Parenteral Nutrition Bags in a Tertiary Referral Hospital: Current Prescribing Patterns

**DOI:** 10.3390/nu10081079

**Published:** 2018-08-13

**Authors:** Beatriz Pelegrina-Cortés, Laura M Bermejo, Bricia López-Plaza, Samara Palma-Milla, Natalia García-Vázquez, Carmen Gómez-Candela

**Affiliations:** 1 Endocrinology and Nutrition Department, University Hospital of Móstoles, 28935 Madrid, Spain; beapelegrina@gmail.com; 2 Nutrition Research Group, Hospital La Paz Institute for Health Research (IdiPAZ), 28046 Madrid, Spain; bricia.plaza@idipaz.es; 3 Dietetic and Clinical Nutrition Department, La Paz University Hospital, Hospital La Paz Institute for Health Research (IdiPAZ), University Autonoma of Madrid, 28046 Madrid, Spain; samara.palma@salud.madrid.org (S.P.-M.); cgcandela@salud.madrid.org (C.G.-C.); 4 Pharmacy Department, La Paz University Hospital, 28046 Madrid, Spain; mnatalia.garcia@salud.madrid.org

**Keywords:** parenteral nutrition, nutritional composition, nitrogen content, lipid emulsion, prescribing patterns, tertiary hospital, acute disease

## Abstract

Individualized parenteral nutrition is the most specialized type of nutritional support in the hospital setting. The composition and prescribing patterns for parenteral nutrition have evolved due to new emerging scientific evidence. In the last few years, there has been a tendency to increase the nitrogen and lipid content and decrease the carbohydrate content. To assess the prescribing pattern in a tertiary referral hospital in Spain, the nutritional composition of individualized parenteral nutrition was evaluated retrospectively from January to December of 2016. A total of 3029 parenteral nutrition units were analysed, corresponding to 257 hospitalized adult patients. Medical specialists in General Surgery and Haematology were the most common petitioners. The three most frequently prescribed parenteral nutrition formulae contained 13.4 (28.8%), 15.7 (19.54%) and 17.9 (17.79%) g of nitrogen. The quantity of carbohydrates and lipids showed a mean non-protein calories-to-nitrogen ratio of approximately 78:1 and a carbohydrate-to-lipid ratio that was near 50:50 in most cases. These results suggest a trend towards the administration of parenteral nutrition with a high content of nitrogen and smaller proportion of the non-protein components.

## 1. Introduction

Individualized parenteral nutrition (PN) is the most specialized type of nutritional treatment in inpatient settings and is received by approximately 2–3% of patients [1].

Since the discovery of circulation by William Harvey in 1628, many scientists have tried to administer nutrients through the parenteral route with favourable and unfavourable results. Of note are the contributions of Stanley Dudrick, who first administered intravenous nutrients directly into the central venous circulation. He produced satisfactory results by first raising beagle puppies fed only this way and later from the first infant fed with only an intravenous nutrient supply in 1967 [2]. Thus, the evolution of science allowed the improvement of this nutritional support, especially in the second half of the 20th century. In the last few years, efforts have been focused on PN solutions and the amounts of macro- and micronutrients added to the bag. In that sense, the increase in the protein content of PN and the evolution of the lipid emulsions has been highlighted. 

Regarding the protein component, a considerable amount of literature has emerged that recommends increasing the amount of nitrogen in the PN bags, which is more important than the total calorie count in some cases [3]. 

The first available intravenous lipid emulsion (IVLE) was developed by Schubert and Wretlind in 1961 and consisted of soybean oil stabilized by egg phosphatides [4]. The soybean lipid emulsions were characterized by a high content of omega-6 polyunsaturated fatty acids (PUFA) [5]. Despite being reasonably safe, the potential pro-inflammatory and hepatotoxic effects of soybean emulsions were well known [6] and they were reconfigured for the latest IVLE generations. These later generations incorporate alternative lipids, such as olive oil or fish oil, to create olive/soy-based formulations. The recent IVLEs use a combination of many lipids (soy/medium chain triglycerides/olive/fish oil), which have anti-inflammatory and immune modulating effects and little hepatotoxicity [7]. SMOFlipid^®^ (Fresenius Kabi^®^), containing soy (30%), medium-chain triglycerides (MCT) (30%), olive oil (25%) and fish oil (15%), belongs to the group of IVLEs with the above-mentioned beneficial properties. 

These two alterations, which are namely the increase in the protein component and the latest IVLE, have changed the macronutrient profile of the PN bags over the last years, leading to the incorporation of more lipids and nitrogen and fewer carbohydrates.

However, data about real clinical practice are scarce, especially data not from Intensive Care Units (ICUs). To assess whether our prescribing patterns comply with the literature recommendations and to review our own protocols, we have studied the composition of all individualized PN bags prescribed by our Nutrition Support Team for adults not staying in the ICU during the year of 2016. 

## 2. Materials and Methods 

A retrospective descriptive study was conducted. Records were collected for the nutritional composition of all individualized PN units, which were prescribed by physicians of the Nutrition Unit of La Paz University Hospital, Madrid, Spain, and aseptically manufactured in the pharmacy of the same hospital as all-in-one solutions between January and December of 2016. The following data were collected: macronutrient profile (nitrogen, carbohydrates, lipids (g)), type of intravenous lipid emulsion, micronutrient profile (sodium, potassium, chloride, calcium, magnesium and phosphorus (mEq/L)), volume (mL) and total calories (kcal). The nonprotein calories-to-nitrogen ratio (NPC:N) and carbohydrate-to-lipid (CH/lipid) ratio were also calculated. Other general variables were collected, including: number of PN units prescribed per patient and the medical speciality of the petitioners. Commercial multi-chamber PN units, paediatric PN (patients <18 years old) units and PN units from ICU stays were not included. 

The physician prescribers have several individualized standard formulae available that were designed together with the hospital pharmacists, which can fit many of the possible patient macronutrient profiles. These formulae consider a range of weights and degrees of metabolic stress and have a fixed composition. If they fit the patient’s calculated requirements, the pharmacists use this particular formula for the PN unit. If the prescribers ask for any modification to the profile, it is made. 

All analyses were performed using SPSS v.17.0 software (SPSS Inc., Chicago, IL, USA). Significance was set at α = 0.05. Descriptive statistics were calculated (mean and standard deviation (SD) for quantitative variables and percentages for qualitative variables).

## 3. Results

**Figure 1:**
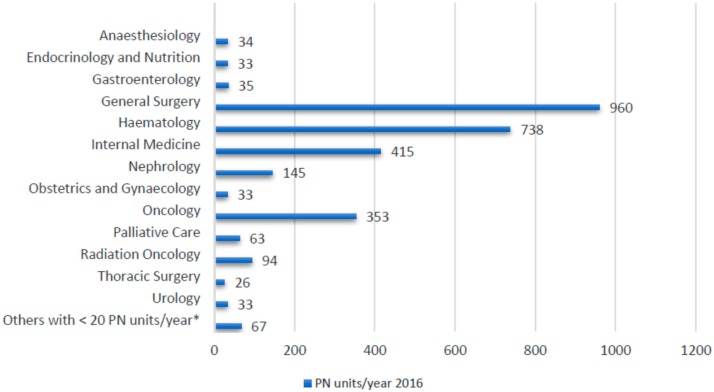
Distribution according to the clinical requesting service. * Others with <20 PN units/year include Cardiac Surgery (9), Cardiology (19), Pneumology (9), Neurology (4), Oral and Maxillofacial Surgery (11), Orthopaedic Surgery (9), Plastic Surgery (3) and Vascular Surgery (3). PN: Parenteral Nutrition.

A total of 3029 individualized parenteral nutrition units, corresponding to 257 patients, were analysed. Each patient received an average of 11.7 PN units during his/her hospital stay. Figure 1 shows the number of PN units distributed by each clinical requesting service. 

### 3.1. Macronutrient Composition

The prescribed PN formulae included various quantities of nitrogen (from 3.8 to 22 g/bag; Table 1). The three most commonly prescribed PN formulae contained the following amounts of nitrogen: 13.4 g (875 units, 28.8%), 15.7 g (592 units, 19.54%) and 17.9 g (539 units, 17.79%).

Carbohydrate (dextrose), lipid and macronutrient ratios are described using the amount of N as a reference (Table 2). In the PN group containing 13.4 g of N, the most common macronutrient profile contained 140 g of carbohydrates with 55 g of lipids (551 units, 18.19%), while the second most common macronutrient profile included 170 g of carbohydrates with 70 g of lipids. Other carbohydrate quantities (120, 150, 160 or 165 g) were added at smaller proportions (<10%). On the other hand, in the PN group containing 15.7 g of N, the most common quantities of carbohydrates were 160 and 200 g with 60 and 80 g of lipids, respectively. Finally, the amount of carbohydrates that was most commonly added to PN containing 17.9 g of N was 180 g with 70 g of lipids, while the second most common addition was 225 g of carbohydrates with 90 g of lipids. The total calorie contents are also shown in Table 2. 

Regarding the lipid profile, all the parenteral nutrition units prescribed in the La Paz University Hospital included SMOFlipid^®^, except for 13 PN units (corresponding to 6 patients), which did not contain any type of IVLE.

### 3.2. Micronutrient Composition and Volume

The micronutrient profile is detailed in Table 3 and volume in Table 4. All PN formulae contained vitamins and trace elements (data not shown).

## 4. Discussion

The quality and quantity of the nutrients in parenteral nutrition formulae have been changing in recent years due to increased knowledge and changes in the recommendations made by scientific organizations. For example, we have increased the amount of nitrogen in the parenteral nutrition formulae and drastically decreased the total calorie content in the last few years.

New data have emerged, particularly regarding critically ill patients, which supports the need for increasing the amount of nitrogen in PN formulae. In 2017, the International Protein Summit recommended a minimum of 1.2 g/kg/day and up to 2.0–2.5 g/kg/day of protein for critically ill patients admitted to the ICU [8]. These protein contents are similar to those suggested in 2016 by the American Society for Parenteral and Enteral Nutrition (ASPEN) guidelines [9] as they observed that the protein requirements may be in the range of 1.2–2.0 g/kg of actual body weight/day, thus driving their recommendation of high protein content. However, the 2009 European Society for Clinical Nutrition and Metabolism (ESPEN) guidelines recommended an amino acid supply of approximately 1.3–1.5 g/kg of ideal body weight/day [10]. Singer et al. [3] also recommended a high protein intake (1.5 g/kg/day) during the early phase of the ICU stay and they highlighted the importance of the nitrogen component regardless of the simultaneous calorie intake. 

Data about the non-critically ill patient (outside of guidelines) are scarce. The ESPEN guidelines advised a dosage that depends on the aetiology of the illness. This can range from a minimum of 1.0 g/kg/day, such as for medical inpatients with multiple comorbidities [11], to a maximum of 2.0 g/kg/day in others, such as some oncology patients [12,13]. The extent of the malnutrition or the severity of the protein depletion will also influence the protein requirements. For example, the ESPEN expert group recommendations for action against cancer-related malnutrition [14] suggest that more than 1.2–1.5 g/kg/day of protein may be needed in severely depleted patients. Additionally, for protein-energy wasting during chronic renal failure, adapting the dietary protein intake target is recommended [15]. A study showing the trend of increasing nitrogen content and reducing NPC:N ratio inside and outside of the ICU was performed by Villalobos-Gámez et al. [16]. They evaluated the nitrogen content of 1604 PN units prescribed in the hospitalization ward and in the ICU of a Spanish tertiary level hospital during a 4-year period. The mean nitrogen content increased from 14.00 ± 3.28 in the first year to 15.05 ± 4.24 in the last one (g of nitrogen, mean ± SD), while there was a decrease in the NPC:N ratio from 111.6 ± 22.2 to 101.8 ± 25.4. For the last year of their study, they also reported the following nitrogen contents (g of nitrogen, mean ± SD) and the NPC:N ratios for the hospitalization ward, the ICU and the subset of ICU patients with renal replacement therapy: 13.56 ± 3.13, 15.87 ± 4.19 and 17.60 ± 4.28, respectively for nitrogen content and 112.2 ± 20.1, 97.3 ± 22.6 and 89.68 ± 18.4, respectively for NPC:N ratio. The mean amount of nitrogen in the hospitalization ward is very similar to our most commonly prescribed amount of nitrogen, but our NPC:N ratios are more similar to those prescribed in the ICU. This is because many of our patients are in an acute phase of their illness, such as patients with haematological tumours who require a stem cell transplantation or those who are in the postoperative phase of major surgery. All of these patients require more nitrogen and less glucose and lipids, resulting in a lower NPC:N ratio. The distribution between the protein and non-protein components is quantitively described to create the PN and confer the desired degree of metabolic stress to the formula, considering that both protein and non-protein calories are oxidized as a calorie source.

SMOFlipid^®^ was first approved in Europe in February 2004 and for the USA in July 2016. Since it became available, many studies have assessed its properties and compared it with other IVLEs. In one study comparing ClinOleic^®^ (MCT/long chain triglycerides (LCT) and oleic acid, Baxter^®^)) and SMOFlipid^®^ in 154 surgical patients who needed PN for at least 7 days, no significant differences were found for the clinical parameters (days on PN, length of stay, infection and mortality) or the biochemical parameters (lipid profile, hepatic profile, glucose, albumin, lymphocytes) between the two IVLEs. The authors attribute the lack of differences to the similar fatty acid compositions of both emulsions (having less PUFA than their previous soy-based lipid emulsion) and the presence of α-tocopherol in both solutions. The lipid content of their PN comprised 40% of the non-protein calories [17]. In 2013, Klek et al. [18] conducted a double-blind, multicentre study on 73 adults with stable intestinal failure who were randomized to receive PN with SMOFlipid^®^ or PN with Intralipid^®^ (soybean oil emulsion, Fresenius Kabi^®^) for 4 weeks. These PN contained a lipid dosage of 1–2 g/kg/day. In the 4th week, they found that the mean concentrations of alanine transaminase (ALT), aspartate transaminase (AST) and total bilirubin were significantly lower in the SMOFlipid^®^ group (*p* = 0.049, 0.027 and 0.043, respectively). They did not find differences in serum triglycerides, haematological parameters, International Normalized Ratio (INR), or cytokine markers of inflammation, such as Interleukin-6 (IL-6), Tumoral Necrosis Factors-RII (_S_TNF-RII) and C Reactive Protein (CRP). Tien et al. [19] evaluated the safety and efficiency of SMOFlipid^®^ compared to other parenteral lipid emulsions in postoperative patients in a meta-analysis of randomized controlled trials, which included 6 trials with a total of 306 patients. Other parenteral lipid emulsions were Lipoven^®^ (soybean based, Fresenius Kabi^®^), ClinOleic^®^ (Baxter^®^) and MCT/LCT^®^ (MCT/LCT, B.Braun^®^). The duration of PN treatment was 4–6 days. Compared to Lipoven^®^ and ClinOleic^®^, SMOFlipid^®^ decreased serum liver enzymes and unlike Lipoven^®^, it decreased low-density lipoprotein triglycerides and CRP levels. They found no differences between SMOFlipid^®^ and MCT/LCT^®^. The studies evaluating SMOFlipid^®^ and Lipoven^®^ only provide moderate quality evidence.

Our hospital first introduced SMOFlipid^®^ in January 2005 and based on the already mentioned beneficial properties, the satisfactory evolution of our patients and the current guidelines, we were able to increase the proportion of lipids in the non-protein component and decrease the amount of glucose. Therefore, our results show that most of our PN have a CH:lipid ratio that is close to 50:50. With this ratio, the patient benefits from lowered glucose and avoids unnecessary overload. On the other hand, as the amount of nitrogen has been calculated based on the patient’s weight and degree of metabolic stress, the lipid composition of the PN does not influence the protein component. These results comply with the 2009 ESPEN guidelines, which recommend the use of formulae with higher lipid contents (up to 50% of the total energy) in geriatric patients [20]. For patients with alcoholic steatohepatitis and liver cirrhosis, they proposed that the glucose amount should cover 50–60% of the non-protein energy requirements with 40–50% of lipids (with IVLE containing less N-6 PUFA than the traditional pure soybean oil emulsions) [21]. For non-surgical oncology patients, they suggest using a higher percentage of lipids, such as an amount of lipids that will cover 50% of the non-protein energy requirements [13]. On the other hand, in 2009, the ESPEN guidelines in Gastroenterology recommended that 2/3 of the calorie requirements should be derived from glucose and 1/3 from a lipid emulsion [22]. For acute pancreatitis, glucose should represent between 50% and 70% of total calories, while the infusion should be temporarily discontinued if persistent (>72 h) hypertriglyceridemia occurs (>12 mmol/L = 1050 mg/dL) [23]. Finally, for surgical patients, they mentioned a tendency to increase the CH:lipid calorie ratio of the non-protein calories from 50:50 to 60:40 or even 70:30 due to the problems with hyperlipidaemia and fatty liver, which is a grade C recommendation [24]. However, in cases of liver damage or persistent elevated liver function tests, we also adapted our PN formulae in order to adjust the recommendations to fit with our clinical practice. This involves changing the amount of glucose (always ≤5 g/kg/day) and lipids (≤1 g/kg/day) and even administering the PN bag for approximately 12 h. Similarly, these actions are also recommended in cases of liver damage that is associated with home PN [25] but these data have not been analysed. In the more recent ESPEN guidelines [5,11,26,27], no specific data about the CH:lipid ratio are provided, except in the case of Cancer Patients Guidelines, where they strongly recommend increasing the ratio of energy from fat to energy from carbohydrates in cancer patients with insulin resistance that have been losing weight [12].

In a recent survey [28] by the ASPEN PN Safety Committee that asked clinicians about IVLE prescription, preparation and administration for adult patients, 42.1% of clinicians prescribed a compounded dextrose/amino acid solution (2-in-1); 41.9% prescribed a compounded total nutrient admixture (3-in-1); 1.7% prescribed a commercial multi-chamber bag with injectable lipid emulsion (ILE) in the bag; 12% prescribed a commercial multi-chamber bag without ILE in the bag; and 2.3% prescribed a commercial multi-chamber bag without ILE in the bag, with the pharmacy adding ILE to the bag. ILE administration as a separate infusion was noted by 43.1% of the surveyed clinicians. This differs from our current clinical practice, where almost 99% of patients receive lipids in their individualised PN made by the pharmacy service (according to our requests) as an all-in-one solution. Only 13 PN nutrition units were formulated without lipids, which reflects the vagaries and need for clinical judgement in clinical practice. Overall, some reasons for the omission of lipids in our clinical practice include moderate-to-severe hepatopathy, such as severe hepatic graft-versus-host disease, sinusoidal obstruction syndrome in stem cell transplantation or severe hypertriglyceridemia.

The micronutrient profiles and volumes show that a commercial multi-chamber PN would not have fulfilled the electrolyte and fluid requirements of our patients, because the average fixed electrolyte profile provides fewer electrolytes, particularly sodium, potassium and chloride, in more volume. Beattie C et al. [29] obtained similar results when they compared 97 individualised PN bags with premixed multi-chamber ones as they observed that the sodium, potassium and chloride concentrations were significantly lower in the premixed bags. These three electrolytes can be the limiting component when prescribing a multi-chamber PN in some acute patients. Physicians from our Nutrition Unit also prescribe this type of PN when it fits the patient needs, particularly the macronutrient needs, and even aseptically add in the pharmacy vitamins, trace elements and electrolytes when the patient requires them. However, these results are not shown as this was not the purpose of the study. 

Our nutrition support team takes care of patients both in outpatient and inpatient settings. In the latter, the nutrition support team conducts an assessment of nutritional status and gives individualized nutritional treatment for patients under our care. The two main applicant services for our parenteral nutrition services are General Surgery, which is not surprising in a tertiary referral hospital with many daily complex surgeries, and Haematology, which has numerous stem cell transplantations each year.

Together with Haematology, we have recently developed a protocol of nutritional intervention to cover the nutritional needs of their patients, who often must be carefully managed as they are critically ill [30]. The suggestions of ESPEN seem to favour enteral tube feeding over PN, unless severe mucositis, vomiting, diarrhoea, malabsorption, ileus or gastrointestinal graft-versus-host disease exists [12]. According to our experience, this symptomatology frequently appears in these transplantation patients and thus, we generally start PN on the 2nd day post-transplantation [30].

## 5. Conclusions

To develop a high-quality and efficient clinical practice that is responsive to the current state of knowledge, it is essential to perform an analysis of results.

The nutritional composition of parenteral nutrition formulae analysed in our study was chosen based on the calculation of the nutritional requirements of each of our patients. This calculation follows the protocols used in our hospital and is in line with the recommendations expressed by various scientific organizations and our experience.

Our results allow us to affirm that we frequently ask our colleagues from the Pharmacy department for PN preparations with a high content of nitrogen in relation to the amount of non-protein calories, which also depends on the degree of metabolic stress of the patient. Using high-quality fat preparations, we will match the amount of non-protein calories from glucose and fat with the long-awaited best metabolic tolerance. Likewise, the individualization of the contribution of micronutrients and volume to be infused is essential. This obviously requires daily monitoring of the patient and continuous adjustment based on the clinical evaluation as it occurs in our clinical practice. In any case, more studies in real clinical practice are needed to guide clinicians in their daily clinical decisions.

Although the use of ready-to-use parenteral formulations is becoming increasingly common, those who design the formulations will have to take the usual hospital practice into consideration as well as the recommendations given by scientific organizations in their clinical practice guidelines.

## Figures and Tables

**Table 1:** Parenteral nutrition units prescribed according to nitrogen quantity.

Nitrogen (g)	PN Units (%)	Nitrogen (g)	PN Units (%)
5.4	124 (4.09)	13	38 (1.25)
6	27 (0.89)	13.4	875 (28.8)
7	36 (1.18)	14.5	55 (1.81)
8	168 (5.54)	15	30 (0.99)
8.4	115 (3.79)	15.7	592 (19.54)
9	84 (2.77)	17.9	539 (17.79)
10	144 (4.75)	20	45 (1.48)
11	61 (2.01)	Others with <10 units ^1^	40 (1.32)
12	56 (1.84)		

^1^ Others with <10 PN units: 3.8 g (0.13%), 4 g (0.06%), 5 g (0.06%), 6.6 g (0.16%), 12.5 g (0.29%), 16 g (0.19%), 18 g (0.13%), 19 g (0.16%) and 22 g (0.09%). PN: Parenteral Nutrition.

**Table 2:** Carbohydrate, lipid and macronutrient ratios along with total calories in prescribed PN formulae using nitrogen content as a reference.

Nitrogen (g)	Carbohydrates (g)	Lipids (g)	CH/Lipid Ratio	NPC:N	PN (Units (%))	Total Energy Intake (kcal)
13.4	140	55	53.08/46.92	78:1	505 (16.67)	1447.49
13.4	170	70	51.90/48.1	97:1	215 (7.09)	1716.01
15.7	160	60	54.23/45.77	75:1	354 (11.68)	1632.51
15.7	200	80	52.63/47.37	96:1	195 (6.43)	1992.51
17.9	180	70	53.33/46.67	75:1	379 (12.51)	1867.5
17.9	225	90	52.63/47.37	95:1	106 (3.49)	2247.5

CH: Carbohydrate. NPC:N: Nonprotein calories-to- nitrogen ratio. PN: Parenteral Nutrition.

**Table 3:** Micronutrient profile in relation to nitrogen content (mean ± SD).

N (g)	Na (mEq)	K (mEq)	Cl (mEq)	Mg (mEq)	Ca (mEq)	P (mEq)
13.4	97.01 ± 25.19	67.97 ± 26.66	80.16 ± 24.21	15.13 ± 3.66	13.99 ± 4.03	22.52 ± 8.24
15.7	84.84 ± 34.13	72.38 ± 25.09	80.01 ± 25.18	14.74 ± 4.39	14.35 ± 4.01	22.76 ± 7.94
17.9	102.87 ± 17.24	71.31 ± 19.29	84.77 ± 14.80	14.70 ± 4.30	14.96 ± 2.08	23.45 ± 8.55

**Table 4:** Total volume of parenteral nutrition in relation to nitrogen content (mean ± SD).

N (g)	Volume (mL)
13.4	1891.92 ± 251.71
15.7	2109.71 ± 297.89
17.9	2443.91 ± 392.19
